# Different profiles and epidemiological scenarios: past, present and future

**DOI:** 10.1590/0074-02760200409

**Published:** 2022-05-20

**Authors:** David E Gorla, Zhou Xiao-Nong, Lileia Diotaiuti, Pham Thi Khoa, Etienne Waleckx, Rita de Cássia Moreira de Souza, Liu Qin, Truong Xuan Lam, Hector Freilij

**Affiliations:** 1 Universidad Nacional de Córdoba, Instituto de Diversidad y Ecología Animal, CONICET, Córdoba, Argentina; 2 Shanghai Jiao Tong University, Chinese Centre for Tropical Diseases Research, National Institute of Parasitic Diseases, One Health Centre, Shanghai, China; 3 Fundação Oswaldo Cruz-Fiocruz, Instituto René Rachou, Belo Horizonte, MG, Brasil; 4 Science Services of Insect Joint Stock Company, Nam Tu Liem district, Ha Noi, Viet Nam; 5 Université de Montpellier, Institut de Recherche pour le Développement, Centre de Coopération Internationale en Recherche Agronomique pour le Développement, Unité Mixte de Recherche, Interactions in the Neglected Tropical Diseases due to Trypanosomatids, Montpellier, France; 6 Universidad Autónoma de Yucatán, Centro de Investigaciones Regionales Hideyo Noguchi, Mérida, Yucatán, México; 7 Institute of Ecology and Biological Resources, Vietnam Academy of Science and Technology, Ha Noi, Vietnam; 8 Hospital de Niños Ricardo Gutiérrez, Servicio de Parasitología y Chagas, Buenos Aires, Argentina

**Keywords:** Chagas disease, epidemiological scenarios, global health

## Abstract

The multiplicity of epidemiological scenarios shown by Chagas Disease, derived from multiple transmission routes of the aetiological agent, occurring on multiple geo-ecobiosocial settings determines the complexity of the disease and reveal the difficulties for its control. From the first description of the link between the parasite, the vector and its domestic habitat and the disease that Carlos Chagas made in 1909, the epidemiological scenarios of the American Trypanosomiasis has shown a dynamic increasing complexity. These scenarios changed with time and geography because of new understandings of the disease from multiple studies, because of policies change at the national and international levels and because human movements brought the parasite and vectors to new geographies. Paradigms that seemed solid at a time were broken down, and we learnt about the global dispersion of *Trypanosoma cruzi* infection, the multiplicity of transmission routes, that the infection can be cured, and that triatomines are not only a health threat in Latin America. We consider the multiple epidemiological scenarios through the different *T. cruzi* transmission routes, with or without the participation of a Triatominae vector. We then consider the scenario of regions with vectors without the parasite, to finish with the consideration of future prospects.

**Vectorial transmission**

*Non-native vector species* - The first scenario targeted by Carlos Chagas himself and then by Chagas disease control programmes was the one that included triatomine species that occupied geographic spaces well outside the one where the species were native, were particularly well adapted to man-made structures and had the highest vectorial capacity for *Trypanosoma cruzi* transmission. This was the situation of most of the Southern Cone countries of South America with *Triatoma infestans* as well as most of Central America, and parts of Colombia and Venezuela with *Rhodnius prolixus*, the two species best adapted to successfully colonise poor quality rural houses and transmit the parasite. Although it seems more or less obvious today, the concept of native and non-native triatomine species was taken into account only after realising that the same control intervention efforts did not produce the same successful outcome for different triatomine species. Indeed, native species maintain sylvatic populations which can act as a potential source of continuous house infestation, thus challenging the success of control strategies directed against domiciliated populations. Starting by the 1950s, the vector control programmes evolved from the use of organochlorinate insecticides, to be replaced by organophosphates, to then adopt synthetic pyrethroids by the 1980. By the time pyrethroids were adopted, recognition was made on the need to improve health education and engage the community to increase the effectiveness of the vector control programs. By that time, an estimated 80% of the infections were attributed to vectorial transmission, and the rest associated to blood transfusion and congenital transmission. By 1991 the onset of the Southern Cone Initiative started the movement of the continental initiatives to control Chagas disease through vector control interventions and blood screening. As a result, the control of blood banks in Latin America successfully eliminated transfusional transmission of *T. cruzi* and vast areas of Latin America are certified as places where the transmission of *T. cruzi* by triatomines living inside houses is interrupted. The most successful non-native species (*T. infestans* and *R. prolixus*) have been eliminated from many parts of the southern cone of South America and Central America, and mainly remain in places corresponding to the geographic range where these species are native, and sylvatic populations contribute to reinfestation.^1^ The vectorial transmission of *T. cruzi* is still active in rural areas of the Gran Chaco, in parts of the Andean region, Central America, Mexico and incipiently recognised in the southern areas of the USA.

A particular scenario for the vectorial transmission of *T. cruzi* is represented by triatomine populations that colonise houses in urban environments, as the well documented cases of Arequipa (Peru),^2^ San Juan city and Pampa del Indio (Argentina),^3^ ^,^ ^4^ by *T. infestans* and *Panstrongylus geniculatus* and *Eratyrus mucronatus* in the Metropolitan District of Caracas (Venezuela).^5^ ^,^ ^6^

*Native species vectors*: s*pecies with populations colonising domestic and peridomestic habitats* - As the relative epidemiological importance of *T. infestans* and *R. prolixus* decreased in settings where they were introduced because of the success of vector control programs, native populations of these species, as well as other species increased visibility and consequently attracted more attention. As mentioned before, the success of indoor residual insecticide spraying against *T. infestans* in most of the Southern Cone countries and *R. prolixus* in Central America was obtained only in settings where populations of these species were exclusively domesticated and introduced (i.e., with no sylvatic populations), which considerably limited the possibilities for re-infestation following spraying. On the other hand, the control of most other triatomine species/populations in their native geographic range has been more challenging, mostly because domestic populations remain connected to peridomestic or sylvatic populations (where residual insecticide applications have low efficacy or cannot be applied), which can then contribute to reinfestation. This explains why, in areas where *T. infestans* peridomestic or sylvatic foci exist, the elimination of house infestation is jeopardised. For example, the persistence and re-infestation of houses by this species in the Andean region can be attributed, at least in part, to the dispersal of bugs from sylvatic populations.^7^ ^,^ ^8^ ^,^ ^9^ Dispersal of these sylvatic bugs towards houses for re-infestation thus need to be taken into account for an effective control.

Another emblematic case is that of *Triatoma dimidiata* in the Yucatan peninsula, Mexico. In this region, the intrusive behaviour of *T. dimidiata*, which infests houses on a seasonal basis from the sylvatic environment surrounding the houses and peridomestic structures, is well described.^10^ In this context, traditional insecticide residual spraying interventions have shown to be unsustainable, while other control strategies, based for example on the use of mosquito nets, have shown promising results and may be incorporated into guidelines to allow for scaling-up.^1^ ^,^ ^11^ ^,^ ^12^ Indeed, this kind of strategies can be adapted in different contexts where *T. cruzi* is mainly transmitted by native species.

The complexity of the problem posed by native species can be exemplified by the great diversity of vector species in Brazil, where the set of measures adopted by the Chagas Disease Control Program started, on a regular basis in 1975, and enabled the International Certification of the Interruption of Vectorial Transmission by *T. infestans*.^13^ ^,^ ^14^ ^,^ ^15^ However, this certification did not represent an end to the risk of vector transmission in the country. Currently, 66 native triatomine species have already been described,^16^ ^,^ ^17^ some of which have intensified their presence in human houses after control of the main vector, representing a major challenge for epidemiological surveillance, since they are not susceptible to elimination.^18^ In this context, the following describes some of the triatomine species that demand greater attention, monitoring by entomological surveillance, and inclusion in health education, in the different endemic regions of the country.

In the semi-arid Caatinga eco-region of northeastern Brazil, *Triatoma brasiliensis* Neiva, 1911 (the main vector) and *Triatoma pseudomaculata* Correa & Espínola, 1964 (a secondary vector) account for a large part of vector-mediated infections, including the states of Ceará and Rio Grande do Norte, where *T. infestans* was never recorded.^15^ ^,^ ^19^ Widely distributed, these vectors are able to colonise both the natural environment and artificial ecotopes, where they are often captured, sometimes even after a short period of insecticide application.^20^ ^,^ ^21^ Among the factors associated with the rapid process of reinfestation and colonisation of households, especially by *T. brasiliensis*, are the proximity of houses to the sylvatic environment, the great plasticity of adaptation to environmental conditions, the transience of insecticides due to a number of causes, and the possibility of operational failures.^22^

*Panstrongylus megistus* (Burmeister, 1835), the first species of triatomine identified as a *T. cruzi* vector,^23^ has the greatest potential for adaptation to domestic environments in Brazil,^24^ being considered the main species of epidemiological importance, excluding the North and North-Eastern regions.^15^ Having its origin in the humid forests of the Atlantic Forest biome,^14^ aridity is the only climatic variable limiting its dispersal.^25^ In the South-East region, especially in the states of Minas Gerais and São Paulo, *P. megistus* is strongly adapted to different environments, including human residences.

Also in the South-Eastern region, the importance of *Triatoma vitticeps* (Stål, 1859) in areas of humid climate and mountainous terrain is apparent. Despite the difficulty of forming domestic colonies, the successive invasion of homes by adult insects from sylvatic foci, with *T. cruzi* infection rates above 60%, highlights the risk of infection.^26^ ^,^ ^27^ ^,^ ^28^ A case of vector-oral transmission reported in the State of Espírito Santo in 2012, where this triatomine is the most captured species, caused the death of a two-year old child.^29^ With similar behaviour, *Triatoma tibiamaculata* (Pinto, 1926),^30^ associated with the Atlantic Forest biome, frequently invades residences in peri-urban areas of Bahia, having been associated with an episode of oral transmission in the State of Santa Catarina.^31^

*Triatoma sordida* (Stål, 1859), the species most captured in Brazil, forms dense colonies in the peridomicile, and, in certain circumstances, it can also colonise the interior of houses.^32^ ^,^ ^33^ It has a wide ecological tolerance that allows it to inhabit several ecotopes and use different blood sources, despite its ornithophilic preference.^34^ ^,^ ^35^ Widely distributed throughout the country, *T. sordida* has its central distribution in areas of the Brazilian Cerrado.^14^ However, anthropic and climatic changes have altered the biocenosis in some places within this biome, triggering a process of aridification, and consequent expansion of the Caatinga to replace the Cerrado, thus promoting the expansion of *T. pseudomaculata* to the detriment of *T. sordida*.^36^

The profile outlined above for each macro-region of Brazil, and the small variety of species mentioned, does not illustrate the full intra-regional diversity, arising from environmental, ecological and social complexities, and political and administrative determinants. The continuous potential for invasion, reinfestation and colonisation of household units from sylvatic foci remains a challenge for maintaining vector control. In this sense, field teams working on entomological surveillance must remain aware of the possibility of persisting residual outbreaks (survival of insects or eggs in places with difficult access and the possibility of resistance to the insecticides used). In time, they should also guide the exposed population on the possibility and risks of invasions by flight of sylvatic triatomines, and to passive dispersion through the transport of firewood, logs, animals, stones, among others, to the interior of houses in endemic regions.^37^ ^,^ ^38^ ^,^ ^39^ However, in the current situation of decentralised surveillance of public health services, many municipalities have shown themselves incapable of conducting regular control actions, both because technical unpreparedness, political lack of interest, and changes in priority due to the emergence of other public health problems.^40^ ^,^ ^41^

*Species with strictly sylvatic populations* - The way in which the human population explores and occupies the environment is strongly related to the dynamics of vector-borne diseases.^42^ For a long time, the Amazon Region was considered to be free of autochthonous Chagas disease,^33^ despite the knowledge about the diversity of native triatomines and sylvatic habits. Special attention was given to specimens of the genus *Rhodnius*, that eventually invade nearby residences, but without evidence of adaptation to the domestic environment.^43^ ^,^ ^44^ Many triatomine species have stable populations only in sylvatic ecotopes, and so the majority are important in the maintenance of the sylvatic cycle of *T. cruzi*. However, several species in this group can transmit the parasite to humans. Some inhabit crown palms nearby human houses, and from there fly into houses to feed. As in many cases infection of the vector with *T. cruzi* is high, the probability of transmitting the parasite could also be high. At least one species does not fly into houses, but inhabit the crowns of industrially important palms, as the case of piassaba palms inhabited by *Rhodnius brethesi*. Collectors of these palms can be infected by *T. cruzi* during their collecting activities. In this context, chemical control strategies to control domestic vectors, which have been successful in countries in the Southern Cone, are not feasible.^33^

**Non vectorial**

The human mobility at different geographical scales has strongly altered the epidemiological scenario of Chagas disease around the world. At the local scale, the migration that started massively by the 1950s from rural to urban areas, brought *T. cruzi* to urban settings of small and large cities within countries in Latin America, outside the geographical range of triatomine vectors. By the end of the 20th century, the migration of human population between countries transported *T. cruzi* to regions where Chagas disease was unknown.^45^ Because of this migration process that occurred at a global scale, people infected with *T. cruzi* are living in virtually all continents. All infected people are able to generate new cases of Chagas disease through blood transfusion, organ transplant and congenitally from mother to unborn child through pregnancy. Blood banks and organ transplant security was one of the first targets of the Southern Cone initiative to interrupt *T. cruzi* transmission for the control of Chagas disease. After the success in the vector control interventions, the congenital transmission appeared as one of the most important route for the production of new infections.

*Congenital transmission* - *T. cruzi* can be transmitted from an infected mother to the child. This transmission route is present in all places where there is an infected pregnant woman, either in a rural or urban Latin American setting, or outside Latin America where no vectors are present. A systematic review of the literature concludes that congenital transmission occurs in 4.7% of the births from infected mothers. With this estimate, between 158.000 and 214.000 children should be tested annually.^46^ ^,^ ^47^ Treating babies below one year of age has one of the highest effectiveness, as therapeutic response is almost 100%, avoiding all future potential damages in the adult life.^48^ Additionally, treatment of girls and/or women during the fertile life prevents the congenital transmission of *T. cruzi*.^49^ The diagnostic algorithm for the pregnant woman and the newborn is well established.^50^ However, weakness of the public health systems allows situation where not all pregnant women are screened for *T. cruzi*, not all newborn are diagnosed, and many children diagnosed as positive are not treated.^51^ It is expected that recent actions from WHO/PAHO through the ETMI+ strategy will improve the implementation of these measures.^52^

*Transfusional transmission* - The transmission of *T. cruzi* through contaminated blood is very well controlled in Latin American countries, but it represents a considerable risk in countries with important immigration from Latin American countries. Studies in Europe and the USA show the burden of this transmission route and the potential consequences of not having a screening system for the management of blood banks.

The transmission of *T. cruzi* through blood and blood products was the second most important route during th 20th century in the Latin American region.^53^ The chance of infection will depend on the amount of blood received, parasite load, and immune status of the receptor. Studies showed that the probability of an infection through contaminated blood ranges between 12-25%.^54^ Control procedures in blood banks are among the most successful interventions of health systems on this aspect of Chagas disease control. This is the result of the continuous improvement of highly sensitive diagnostic kits^55^ and high commitment by the disease control program of the countries. At present, 21 countries in Latin America study the blood of 100% of donors. Outside Latin America, infection cases by blood transfusion have been recorded especially in Spain and Italy, where specific directives for *T. cruzi* detection in blood banks have been established during the last years. However, in other countries of the European Community there are no such specific directives.^56^ ^,^ ^57^ In the United States there are specific directives on this issue elaborated by the Centre for Disease Control and Prevention (CDC) (https://www.cdc.gov/bloodsafety/basics.html).

*Organ transplants* - Would a *T. cruzi* infected person in a chronic phase need an organ transplant, a challenging situation must be faced because of the necessary immunosuppression, that would lead to an infection reactivation. This process would depend on the immunosuppression degree, i.e., in a cardiac transplant it is 30-60%, whereas in a kidney transplant it is 15-25%. On this occasions, a rapid diagnostic is fundamental for a positive evolution.^58^ Would a seronegative person receive a *T. cruzi* infected organ, the prospect would depend on the transplanted organ and the receptor immunosuppression. In any case, the expected mortality is high,^59^ and it is always important to evaluate the serology of the donor. Normative regulation for organ transplant has fundamental importance to avoid *T. cruzi* infection through transplants. Argentina has a normative,^60^ the USA published the normative in 2011,^59^ as well as some European countries, like Italy and (partially) Spain.^61^ In the Latin America region, 13 countries part of the DONASUR defined the protocol for the management of organ transplant (https://www.paho.org/hq/index.php?option=com_content&view=article&id=15680)

*Oral transmission* - Although the oral transmission of *T. cruzi* was described in 1965,^62^ received increased attention from the late 1990s, as the vectorial transmission route decreased its relative importance in many Latin American regions. Typical cases of oral transmission frequently produce micro-epidemics associated with the consumption of natural juices. Food contamination by *T. cruzi* was described by two mechanisms, one associated with the vector and the other with contaminated secretions of a mammal host.^63^ The preparation of home-made acai juice for family commemorations and sugar cane juice offered to tourists on the Atlantic beach are well known cases that occurred during the last 20 years. So far, the scenario of oral transmission episodes appeared mainly in territories that are part of the Amazon ecosystem but occurred outside the Amazon Region as well, and triggered strong recommendations within the area of food security.^64^

**Risks**

*Regions with triatomine vectors with no T. cruzi* - Although the focus of studies on Triatominae has been the region of the Americas because of their relevance as vectors of *T. cruzi*, the presence of Triatominae species outside Latin America is long known. *Triatoma rubrofasciata* is the only cosmopolitan Triatominae, and seven *Triatoma* species occur exclusively outside Latin America. Six species of the *Linshcosteus* genus occur exclusively in India. The Old World Triatominae poses biological and epidemiological questions. In biological terms, they challenge our understanding of the Triatominae evolution and distribution.^65^ Old World Triatominae have recently received increased attention in South East Asia (particularly southern China and Vietnam), as the result of increased field work effort to better understand their distribution and epidemiological importance for the region.

Reports on the presence of *T. rubrofasciata* in Asia has increased in recent years, having been reported in China,^65^ ^,^ ^66^ ^,^ ^67^ Vietnam,^68^ ^,^ ^69^ India^70^ and Sri Lanka.^71^ The reports that showed triatomine findings as a rare event, are now updated and show that triatomine occurrence is a frequent event. A study that collected triatomines using a questionnaire to the Chinese CDC staff and village doctors and social media tools (Wechat and QQ) was carried out from July 2016 to December 2018.^96^ A training course was given to the staff of province-level CDC to identify the species of the samples and unified quality. Time, location, development stage and gender of the samples were recorded, respectively. Meanwhile, light traps^72^ and Noireau traps^73^ were used to collect triatomines in the field. All the samples and data were sent to the National Institute of Parasitic Diseases (NIPD), China CDC for further checking and aggregating. The morphological identification of the triatomines was conducted referred to Xiao et al.^74^ and Lent and Wygodzinsky.^75^ During this survey, 1042 triatomines were collected (854 adults and 188 nymphs), in 170 different sites of 67 counties in 30 cities from the provinces of Guangxi, Guangdong, Fujian and Hainan (Fig. 1). All specimens were identified as *T. rubrofasciata* (Fig. 2C-D) except one adult female (Fig. 2A-B) that was captured in the Dali Prefecture (Yunnan Province). This specimen was different to *T. rubrofasciata* (De Geer, 1773) and *Triatoma sinica* (Hsiae, 1979), showing darker colour and no obviously orange-red or orange-yellow marking, 1st antennal segment not exceeding tip of head, smaller and shorter synthlipsis. Its scutellum was rough and wrinkled, the top of it was slightly upwarping, oblique uplift in the centre of both sides (Fig. 1A-B). The conserved 16S ribosomal RNA gene, the Cytochrome b gene and 28S ribosomal RNA showed 93.08%, 77.16% and 91.55% similarities to *T. rubrofasciata* in GenBank, respectively. The sequences obtained from 16S rRNA of the Dali species were submitted to GenBank under the accession number MN200191. The phylogenetic trees were constructed using the 16S rRNA gene of the Dali species and other triatomines in GenBank and were based on the neighbor-joining method by MEGA6.0 (Molecular Evolutionary Genetics Analysis Version 6.0). The result showed that the Dali species fits in the same clade of *T. rubrofasciata* (Fig. 3). Morphological and molecular data suggest that it could be a novel Triatominae species. *T. rubrofasciata* specimens were collected mostly (1035/1041) inside human houses (60.9%) and domestic animal shelters. Most of the specimens (780/1041) were captured between July and August.

**Fig. 1:**
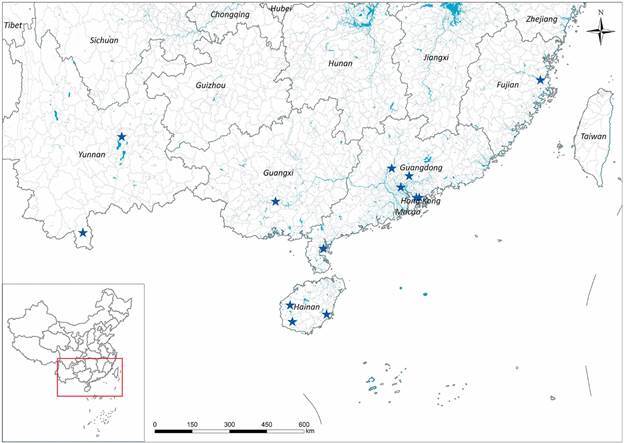
the distribution for *Triatoma rubrofasciata* in China before 2016 (left) and updated with studies by Liu and Zhou after 2016 (right). The map data is from the national basic geographic information centre (http://www.ngcc.cn/ngcc).

**Fig. 2:**
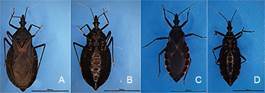
the novel species of *Triatoma* sp. captured in Dali, Yunnan province (A back B ventral), *T. rubrofasciata* captured in Shunde, Guangdong province (C back D ventral).

**Fig. 3:**
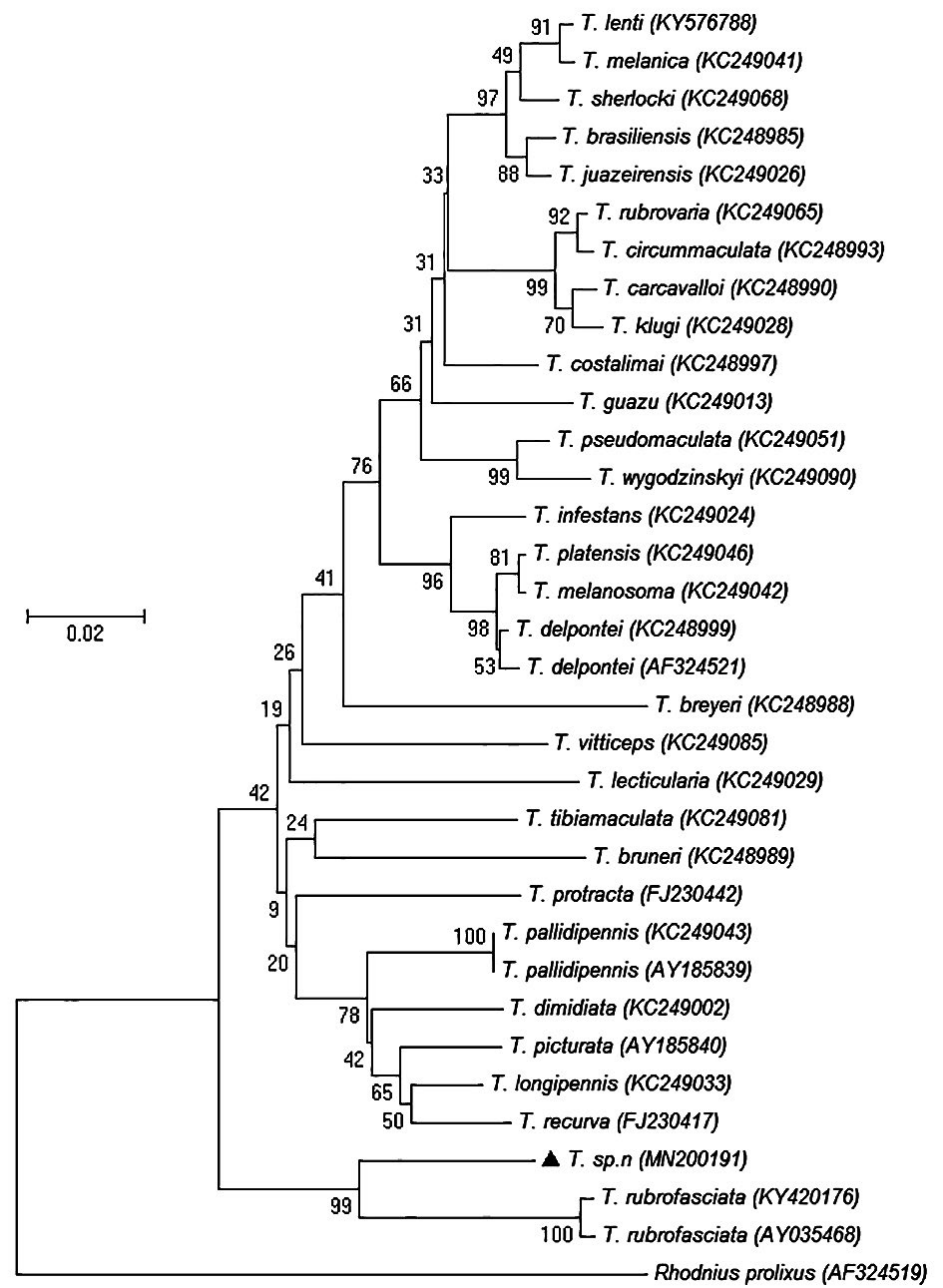
phylogenetic tree showing the relationships between the Dali species (MN200191) and other Triatominae species.

Historical studies reported three Triatominae species from Vietnam: *T. bouvieri*, *T. migrans* (in Ho Chi Minh City) and *T. rubrofasciata* (in 21 districts in Hanoi, especially in Tu Liem and Gia Lam). A national survey during the period 2010-2012 showed that *T. rubrofasciata* is found in at least 21 provinces.^68^ Urban infestations involve hundreds of insects per house, with a seasonal peak of adult bugs from June to September. Although *T. rubrofasciata* from Asia is not infected with *T. cruzi*, it is frequently feeding on humans, causing severe bite reactions that sometimes lead to anaphylactic shock.^68^ ^,^ ^76^ ^,^ ^77^ In recent years, reports of bites of *T. rubrofasciata* to the human population have increased significantly in different places of Vietnam, becoming a public health problem.^68^ ^,^ ^69^ Bitten people present swelling and itching at the site of the bite, sometimes with a local skin infection.^78^ In Hanoi, 24% of the cases also involved a severe fever that lasted between one and two days.^69^

In central Vietnam, *T. rubrofasciata* is present in domestic environments and their surroundings, and apparently absent in wild habitats, despite the various searches that were carried out. Until now, it has never been reported in wild habitats in both the Americas and Asia.^79^ In big cities, this species inhabits houses of good construction quality, with people as the main food source. In these cases, the insects are concentrated in the lower floors of the buildings, probably due to their limited flight capacity. In peridomestic habitats, both in urban and rural areas, *T. rubrofasciata* has always been associated with wood, both in firewood storage places and walls or ceilings of human constructions. All stages of the bugs were collected throughout the year revealing that their reproductive activity is not restricted to a particular season.^78^ *T. rubrofasciata* is an invasive species which, despite its large size and its irritating saliva, seems to be increasing its association with humans. In Vietnam it is already a matter of concern because of its bites and the bite reaction that can ensue. With increased migration of human populations and further development of anthropophilia of *T. rubrofasciata*, it seems entirely possible that *T. cruzi* infections reaching Asia could be picked up by urban *T. rubrofasciata*.^68^

*Regions without triatomines* - The increase of international human mobility represents a real risk of passive transport of triatomines. There are evidences that it happened in the past, and it can happen in the future. The proven colonisation of Central America by *R. prolixus* travelling from Venezuela to France to El Salvador at the beginning of the 20th century produced uncountable number of infections by *T. cruzi*.^80^ There are numerous undocumented instances of voluntary triatomine transport by planes among Latin American and European countries, at least during the 20th century.^81^ After the case of *R. prolixus*, there are no new reports of colonisation by triatomines in regions outside Latin America during the 20th century, but this can be just a matter time. The colonisation of regions near port areas by *T. rubrofasciata*, establishing stable populations is another evidence of this real possibility of triatomine colonisation outside Latin America.^68^

**Looking into the future**

*Surveillance, big data and information technology* - International health agencies, NGOs, donors and all stakeholders that have to decide on resource distribution, need an information system with the ability to assess the status and to show the impact of Chagas disease management at a global scale. For a disease with the complexity shown by Chagas disease the development of such a surveillance system proved to be a difficult task. Presented conceptually at the launch of the Global Network of Chagas Disease in 2007, its initial configuration was defined in 2012. At present, the system is at a testing phase and considers the incorporation of data from country reports plus white and grey scientific literature. The components of the system include data on vectors (occurrence and control interventions), disease prevalence and treatment with the available parasiticide drugs (Bz and Nx), through the multiple data source channels, disparate formats and data aggregation. The system started as the World Information System for Chagas Disease (WISCD) but evolved to become the World Information System for the Control and Erradication of Neglected Tropical Diseases (WISCENTD). Its main objective is to provide national health agencies, researchers and other interested parties, a unified view of the current status of and estimation of future trends on Chagas disease around the world. A brief description is presented at http://mss4ntd.essi.upc.edu/wiki/.

*Disease: detection and treatment* - One of the main problems posed by Chagas disease is that it has a very long silent phase and it is frequently silenced, because of the lack of active search to detect infections. The challenge for the future if to increase the effort to detect and to treat infections. Beside the activities promoted by governmental agencies, the participation of NGOs involving groups of affected people around the world (like FINDECHAGAS for example http://findechagas.org/) showed the importance and impact of the community involvement for the control of Chagas Disease.

*Vector: presence, control efficacy, community involvement, insecticide resistance* - The presence of vectors inside houses was considered the first indication of the parasite transmission risk. The initial activities of Chagas Disease Control Programs targeted the elimination of vectors within houses. In all Latin American countries with affected areas, the presence of vectors within houses is one of the main vectorial transmission risk indicators, except in areas such as the Amazon Region, where transmission occurs due to sylvatic specimens invading houses, and/or through the oral route. House infestation is one of the main indicators considered in the process of certifying the interruption of vectorial transmission, although its low sensitivity is long known, as shown by Abad-Franch et al.^82^ One of the main challenges for the future is to develop a reliable house infestation indicator, better than the one based on the traditional active search, especially under low-abundance vector populations, typical of periods after vector control interventions with residual insecticides. A number of alternatives have been considered, as lured traps,^83^ adhesive tapes,^84^ active search with repeated observations to deal with imperfect detection,^85^ although none were as yet incorporated into routine protocols of vector control programs. More recently, attempts appeared to develop citizen science initiatives to involve the community through the development of mobile applications [GeoVin (http://geovin.com.ar/), TriatoKey (http://triatokey.cpqrr.fiocruz.br/)].

*Vector control innovation. Insecticide impregnated nets (bedroom walls, animal enclosures), new insecticide formulations* - Although the relative importance of vectorial transmission has strongly decreased during the last 30 years, vector control is still an unsolved problem in several parts of Latin America. The problems are related to the lack of sustainability of vector control programs, low sensitivity of house infestation indicators, low efficacy of currently used insecticides against non-domesticated populations and insecticide resistance. Except for the lack of sustainability related to political decisions on resource assignments, the other aspects are technical issues that have to be dealt using a sound science approach.

The certification of vectorial transmission interruption is based, among other aspects, on the demonstration that house infestation by triatomines is lower than 1%. The measurement of house infestation is based on active search, known to have low sensitivity, but widely accepted as the standard. When vectorial transmission interruption is certified in a given area, there is a high chance that the relevance of Chagas disease will decrease importance in the political agenda. The situation could lead to a decrease in assigned resources to disease vigilance, and potentially to a recrudescence of the vectorial transmission. Reliable indicators for house infestation, especially under low vector abundance populations are needed. Besides timed-manual search, several indicators have been considered (community notification, sensor boxes, adhesive tapes, lured traps, repeated timed-manual search). Some comparative studies were made, although there is no information on the consistency of the indicators for different areas in Latin America.

Since 1997, when Uruguay was certified as the first country that interrupted the transmission of *T. cruzi* by *T. infestans*, a number of Latin American countries and/or regions have been certified as places where the vectorial transmission of *T. cruzi* is interrupted. This is certainly a recognition of the efforts by the national health agencies that successfully deployed a vector control program. However, vectorial interruption sustainability is unsolved challenge because of what is now known as the paradox of the punishment of success.^85^

Despite the recognised epidemiological importance of secondary vectors, and the impracticality of eliminating native vectors, in 2012 the PAHO held a workshop to debate a proposal for the “Certification of interruption of the transmission of Chagas disease by native vectors in Brazil”. However, given knowledge of the peculiarities of the epidemiology of the disease, with its silent clinical manifestations and difficulties in early diagnosis and treatment, it would have been irresponsible to grant to the country such recognition. In addition, it must be admitted that the absence of organised epidemiological surveillance makes it impossible to infer the scenario that approximates the real situation of risk in the country. Such certification would result in a total absence of resources for vector control, further weakening of the surveillance system, and, consequently, in the misleading assertion of the absence of new cases of *T. cruzi* transmission.^85^

Insecticide residual spraying with synthetic pyrethroids has been very successful for the control of intradomestic infestation by triatomines in Latin America. However, the efficacy of the same pyrethroids and formulations showed much lower efficacy for the control of triatomine populations occupying peridomestic structures and/or against the invasion of vectors from the sylvatic surroundings. The low efficacy of these interventions is related to the rapid degradation of the pyrethroid molecule under the action of several environmental factors, mainly UV exposure.^86^ An increase in the vector control interventions has been studied, using microencapsulated insecticide formulations,^87^ ^,^ ^88^ treated nets on animal enclosures,^89^ treating peridomestic animals with different active ingredients and/or pyrethroid formulations (pour-ons, spot-ons, powder^21^ ^,^ ^90^ ^,^ ^91^) biological control,^92^ ^,^ ^93^ ^,^ ^94^ habitat modifications.^95^ As peridomestic structures are very different in different areas of Latin America, we can expect that different intervention types would have different efficacy in different regions. There is a need to evaluate and compare the efficacy of the interventions. As peridomestic structures would probably be strongly linked to cultural uses, intervention considering local cultures would have a better prospect for adoption, as shown by the modification of the construction of goat corrals in the arid chaco of Argentina, currently with expanded adoption by rural communities.^95^ Resistance to pyrethroid insecticides and associated vector control failures in triatomines is limited to *T. infestans* populations of northern Argentina and south-eastern Bolivia. It is a highly important aspect of vector control that has to be addressed within the routine activities of vector control programs. Details of ongoing activities are analysed in another chapter of this book.
